# Snowden’s Binaural Stethoscope

**Published:** 1881-02

**Authors:** 


SNOWDEN'S BINAURAL STETHOSCOPE.—We have carefully studied and practiced the use of auscultation privately, and publicly, as a teacher for many years, in the diagnosis of diseases of the chest, on the gravid uterus, in the movements of the circulation, etc., ctc., and have brought into requsition most of the instruments in use, as aids to the auditory organs; but we have never met with any apparatus that has given so much satisfaction, for the purposes mentioned as the improved Binaural Stethoscope, constructed by Mr. William Snowden, of Philadelphia. Our friends credit us with some appreciation of "the concord of sweet sounds," and if a somewhat cultured ear can be so much aided by this instrument, it ought to be of the greatest advantage to those who do not appreciate sounds so easily or naturally.

